# Isolated anti-Ro/SSA thrombocytopenia: a rare feature of neonatal lupus

**DOI:** 10.11604/pamj.2015.22.312.6839

**Published:** 2015-11-27

**Authors:** Imene Dahmane Ayadi, Emira Ben Hamida, Mohamed Riadh Boukhris, Ahlem Bezzine, Sihem Chaouachi, Zahra Marrakchi

**Affiliations:** 1 Department of Neonatology, Charles Nicolle Hospital, Tunis-El Manar University, Tunis, Tunisia

**Keywords:** Neonatal lupus erythematosus, thrombocytopenia, anti-Ro/SSA

## Abstract

We report a rare case of isolated thrombocytopenia related to anti-Ro/SSA antibodies. The mother was followed for unlabeled familial thrombocytopenia. The mother had positive anti-Ro/SSA antibodies. She was asymptomatic without skin lesions or other criteria neither of systemic lupus erythematosus nor other connective tissue disease. Pregnancy was uneventful. The postnatal examination was normal. On the first day of life, blood cells count showed thrombocytopenia at 40 x 10^9^/L. Within the second day of life, platelet level dropped to 20 x 10^9^/L. The management of thrombocytopenia included platelet transfusion and human immunoglobulin infusion. On the fifth day of life, there has been a drop in platelet count to 10 x 10^9^/L requiring renewed platelet transfusion and human immunoglobulin infusion. On the 10^th^ of life platelets rate was stable around 60 x 10^9^/L. The infant had no evidence of cardiac, dermatologic or hepatobilary involvement initially or throughout follow up.

## Introduction

Neonatal lupus erythematosus (NLE) is an uncommon autoimmune disease (incidence 1/20000 live births) [1], frequently undiagnosed. It is a passive autoimmune disease secondary to the transplacental transfer of anti-Ro/SSA, anti-La/SSA and less frequently anti-RNP antibodies to the foetus. NLE includes clinical and laboratory manifestations, whatever the mother is suffering from a systemic autoimmune disease or is totally asymptomatic. Mothers are frequently asymptomatic when NLE is diagnosed [2]. During pregnancy, anti-Ro/SSA antibodies cross the placenta from the 12^th^ week of gestation and persist for 6 to 8 months after birth. The major manifestations are skin lesions and congenital heart block. Minor manifestations include hepatic dysfunction and hematological abnormalities [2]. The pathogenesis NLE is still unclear. The association of NLE manifestations with maternal autoantibodies, which resolve within 6 months after birth with the clearance of the maternal antibodies, is a strong indicator of the importance of autoantibodies for determining the pathogenesis of NLE [3]. In fact, the presence of autoantibodies is not sufficient. Additional risk factors are required for disease development. The amount of maternal antibodies, the anti-Ro/SSA avidity antibodies, and genetic and maternal factors have been implicated.

## Patient and observation

A term male neonate born to a 39-year-old mother with a history of fetal loss, one was with hydropsfetalis. The mother was followed for unlabeled familial thrombocytopenia (her mother, her sister and her brother were affected). There was no family history of autoimmune disease. The investigation of the mother showed positive anti-Ro/SSA antibodies. The mother was asymptomatic without skin lesions or other criteria neither of systemic lupus erythematosus nor other connective tissue disease. The two elder children (a 9-year-old son and a 7-year-old daughter) were healthy and they had not presented thrombocytopenia in the neonatal period. Pregnancy was followed in Charles Nicolle Hospital, a tertiary care centre, it was uneventful. The mother had moderate thrombocytopenia about 80 x 10^9^/L. The delivery was vaginally. The adaptation to extra uterine life was good. The postnatal examination was normal. On the first day of life, blood cells count showed thrombocytopenia at 40 x 10^9^/L. In this context, autoimmune thrombocytopenia was the first etiology evoked, inherited thrombocytopenia was also discussed. Test results of the newborn showed high levels of anti-Ro/SSA antibodies. As part of the investigation of an early neonatal thrombocytopenia, platelet phenotypage showed compatibility between the father, the mother and the newborn. Within the second day of life, platelet level dropped to 20 x 10^9^/L. The newborn has received platelet transfusion, and human immunoglobulin infusion of 1 g/Kg/day for two consecutive days with rise of platelet count to 43 x 10^9^/L. The cranial ultrasound, abdominal ultrasound and ocular funds concluded to the absence of hemorrhage. The electrocardiogram and the echocardiogram were normal. The liver function tests noted the absence of hepatic cytolysis and cholestasis. On the fifth day of life, there has been a drop in platelet count to 10 x 10^9^/L requiring renewed platelet transfusion and infusion of human immunoglobulin. The newborn was discharged on the 10^th^ day of life, when there has been a stabilization of platelets rate around 60 x 10^9^/L. No lupus dermatitis, cardiac or hepatobiliary involvements have appeared during follow-up. Platelet count became normal within the fourth month of life.

## Discussion

We report a rare cause of neonatal thrombocytopenia, due to transplacental transfer of maternal anti-Ro/SSA antibodies. The anti-Ro/SSA antibodies are generally associated with some connective tissue disease especially systemic lupus erythematosus and Sjogren's syndrome, but also some undifferentiated connective tissue diseases. They can also be present in healthy persons. Their estimated prevalence in the general population varies between 0 and 11% [4]. The risk of having a baby with NLE among unselected anti-Ro/SSA antibody-positive women is about 1-2% [1]. The major clinical findings in NLE are cardiac heart block, coetaneous manifestations, hepatobiliary dysfunction, hematologic abnormalities, and exceptionally neurological impairment [2]. The incidence of noncardiac features of NLE has rarely been studied. Few studies have focused on determining the profile of hematological disorders caused by anti-Ro/SSA antibodies. Authors have reported hematologic disorders in about 10% of infants with NLE [3], consisting of neutropenia, thrombocytopenia [5], rare cases of aplastic anemia or hemolytic anemia [6, 7]. A Japanese study, including 193 cases of neonatal lupus, reported hematological disorders in 28 cases (14%), 15 cases (7.5%) had presented isolated thrombocytopenia [8]. A Chinese study, including 94 cases of neonatal lupus, noted 28 cases of thrombocytopenia (30%) [9]. A multicenter prospective study (Toronto, Canada and Milano, Italy), had determined the clinical features of 128 newborns born from 124 pregnancies in 112 women with positive anti-Ro/SSA antibodies, it found a high incidence of hematologic abnormalities (27%), the most frequent anomaly was neutropenia then anemia, thrombocytopenia was present in only 4% of cases. They also noted that newborns of asymptomatic mothers with positive anti-SSA/Ro antibodies represented 12% (4/33) of infant with hematological abnormalities [5]. Neonatal thrombocytopenia secondary to the transplacental passage of anti-Ro/SSA maternal antibodies is rare. It occurs very infrequently as the only manifestation of NLE [10], as it was in our observation. In fact, hematological abnormalities are often under diagnosed because almost always asymptomatic. It is a transient phenomenon; symptoms are reversible and typically disappear within 6 months, in parallel with declining antibodies levels [3].

## Conclusion

In the reported case, thrombocytopenia was the only feature of NLE, which is rare. Given the severity of thrombocytopenia and family history, inherited thrombocytopenia was raised. Thrombocytopenia was corrected in the 4^th^ month of life, concomitant to maternal antibodies purification, excluding this hypothesis.
